# Teneurins: An Integrative Molecular, Functional, and Biomedical Overview of Their Role in Cancer

**DOI:** 10.3389/fnins.2018.00937

**Published:** 2018-12-11

**Authors:** Boris Rebolledo-Jaramillo, Annemarie Ziegler

**Affiliations:** Center for Genetics and Genomics, Facultad de Medicina, Clínica Alemana Universidad del Desarrollo, Santiago, Chile

**Keywords:** Teneurin/ODZ, somatic aberrations, cancer signaling pathways, data mining, tumorigenesis

## Abstract

Teneurins are large transmembrane proteins originally identified in *Drosophila*. Their essential role in development of the central nervous system is conserved throughout species, and evidence supports their involvement in organogenesis of additional tissues. Homophilic and heterophilic interactions between Teneurin paralogues mediate cellular adhesion in crucial processes such as neuronal pathfinding and synaptic organization. At the molecular level, Teneurins are proteolytically processed into distinct subdomains that have been implicated in extracellular and intracellular signaling, and in transcriptional regulation. Phylogenetic studies have shown a high degree of intra- and interspecies conservation of Teneurin genes. Accordingly, the occurrence of genetic variants has been associated with functional and phenotypic alterations in experimental systems, and with some inherited or sporadic conditions. Recently, tumor-related variations in Teneurin gene expression have been associated with patient survival in different cancers. Although these findings were incidental and molecular mechanisms were not addressed, they suggested a potential utility of Teneurin transcript levels as biomarkers for disease prognosis. Mutations and chromosomal alterations affecting Teneurin genes have been found occasionally in tumors, but literature remains scarce. The analysis of open-access molecular and clinical datasets derived from large oncologic cohorts provides an invaluable resource for the identification of additional somatic mutations. However, Teneurin variants have not been classified in terms of pathogenic risk and their phenotypic impact remains unknown. On this basis, is it plausible to hypothesize that Teneurins play a role in carcinogenesis? Does current evidence support a tumor suppressive or rather oncogenic function for these proteins? Here, we comprehensively discuss available literature with integration of molecular evidence retrieved from open-access databases. We show that Teneurins undergo somatic changes comparable to those of well-established cancer genes, and discuss their involvement in cancer-related signaling pathways. Current data strongly suggest a functional contribution of Teneurins to human carcinogenesis.

## Introduction

Teneurins compose a family of large transmembrane proteins identified over two decades ago in *Drosophila* (Baumgartner et al., 1994; Levine et al., 1994). Their high degree of interspecies conservation was evidenced through early phylogenetic analyses, and in the human genome, four highly related gene paralogues (*TENM1* through *TENM4*) were predicted based on DNA sequence homologies and partial expression data (Minet and Chiquet-Ehrismann, 2000; Tucker et al., 2012). In vertebrates as in other species, expression of Teneurins has consistently been allocated to the developing nervous system, where they guide formation of neuronal networks through homophilic and heterophilic interactions across synaptic spaces (Hong et al., 2012; Beckmann et al., 2013; Boucard et al., 2014; Berns et al., 2018). Avian and invertebrate Teneurins have further been related to the development of non-neural tissues including the gonads, heart, pharynx, limbs and the gut, among others (Tucker et al., 2001; Drabikowski et al., 2005; Trzebiatowska et al., 2008). In terms of gene organization, Teneurins have shown to be large and complex, displaying multiple alternatively spliced forms in all species analyzed (Tucker et al., 2001, 2012; Lossie et al., 2005; Berns et al., 2018). Accordingly, Teneurin functional analysis is not straightforward and requires experimental consideration of multiple protein subspecies, which might present distinct spatial and temporal expression patterns during development. This inherent complexity challenges the study of newly discovered Teneurin structural and genetic variants. However, the prominent technological and bioinformatics development, together with the ease of access to molecular information gathered into large cancer databases, facilitates oncology research, and can be applied to understanding the significance of Teneurins in carcinogenesis. To this end, the current review integrates tumor-derived genomic and transcriptomic data available through the literature and open-access repositories. The main subjects have been organized according to the historic timeline of their appearance, with the goal to construct a role of Teneurins in cancer starting form a structural point of view into the function and biomedical associations. Based on current knowledge, we propose a plausible role for Teneurins in tumor cell signaling and as recurrent targets of somatic alterations. The function of Teneurins in neural development will not be explicitly addressed, as it will be discussed in detail in other contributions within the current issue.

## Teneurin Genes: Targets For Structural Alterations in Cell Lines and Tumors

### Chromosomal Rearrangements in Reports and Data Repositories

Since their discovery over two decades ago, Teneurin research has predominantly addressed their importance as morphogens and determinants of neural connectivity during embryonal development. Intrinsically, this has determined the use of suitable animal models such as *C. elegans, Drosophila*, chicken and mice, that allowed functional and structural characterization of Teneurins and analysis of gene expression patterns (Baumgartner et al., 1994; Oohashi et al., 1999; Rubin et al., 1999; Fascetti and Baumgartner, 2002; Drabikowski et al., 2005). Conversely, studies assessing the role of human Teneurins were less frequent and their corresponding gene organization was largely predicted through homology-based sequence alignments and partial cloning strategies (Minet et al., 1999; Ben-Zur et al., 2000; Beckmann et al., 2011; Tucker et al., 2012). Discoveries comprising the human orthologs have thus been belated. This includes the identification of tumor-associated somatic changes involving the Teneurins, which has occurred incidentally and is less evident in the literature. In this context, one of the first traceable publications described a chromosomal translocation involving the *TENM4* and *NRG1* (Neuregulin-1) genes in a breast cancer cell line (Liu et al., 1999; Wang et al., 1999). Importantly, this rearrangement generated a biologically active fusion protein (γ-heregulin) that acted as a secreted autocrine and paracrine growth factor for MDA-MB-175 and MCF-7 breast cancer cells, respectively (Schaefer et al., 1997). Some 10 years later, a second report described recurrent translocations affecting the *IGH* (Immunoglobulin Heavy Locus) and *TENM2* genes in mucosa associated lymphoid tissue (MALT) lymphomas of the skin and the ocular adnexa (Vinatzer et al., 2008). This seems consistent with the role of Teneurin-2 in development of binocular circuits in the visual system (Rubin et al., 2002; Young et al., 2013), while the *C. elegans* ortholog (Ten-1) is essential for hypodermal development (Mörck et al., 2010). A third translocation involving *C11orf73* (current gene name is *HIKESHI*) and *TENM1* was detected in an advanced B3 thymoma (Petrini et al., 2013).

Although scarce, the above findings pose the question whether rearrangement of Teneurin genes is a frequent and biologically relevant event in tumorigenesis. The advent of large, open-access data repositories gathering cancer-related data from thousands of patients, can now partly overcome the lack of explicit reports. Upon a search for chromosomal alterations in two curated sites (ChimerDB 3.0, http://203.255.191.229:8080/chimerdbv31/mindex.cdb, and “Atlas of Genetics and Cytogenetics in Oncology and Hematology”, http://atlasgeneticsoncology.org/) (Huret et al., 2013; Lee et al., 2017), we were able to retrieve additional chromosomal rearrangements involving the *TENM1, TENM2*, and particularly *TENM4* genes (Table 1). Some preliminary observations can be drawn from this overview. First, most reported translocations are derived from a few large and rather recent studies. Earlier data not submitted to a repository might have been missed, as exemplified by the *IGH/TENM2* and *C11orf73/TENM1* translocations in MALT and B3 thymoma, respectively. The frequency of Teneurin rearrangements might thus be underrepresented. Second, most translocations occur with distinct fusion partners and as unique events, both within each tumor type and among different tumors. Third, rearrangements involving *TENM4* clearly predominate (24/32, 75%), followed by *TENM2* (5/32, 15.6%) and *TENM1* (3/32, 9.4%). No translocations have apparently been reported for *TENM3*. Finally, 18/32 (56%) of translocations occurred in breast cancer, and most were intrachromosomal (22/32, 69%).

**Table 1:** Reported tumor cytogenetic rearrangements involving Teneurin genes.

**Rearrangement**	**Source**	**Frame**	**Tumor Site**	**References**
***TENM1***				
t(X;16)(q25;q23) *TENM1/TMEM170A*	RNA (TCGA)	In-frame	Prostate adenocarcinoma	Huret et al., 2013; Yoshihara et al., 2015; Lee et al., 2017
t(X;21)(q25;q21) *BACH1/TENM1*	RNA (TCGA)		Kidney adenocarcinoma	Huret et al., 2013; Yoshihara et al., 2015; Lee et al., 2017
t(X;17)(q25;q11) *KSR1/TENM1*	RNA (TCGA)	5′UTR-CDS	Acute myeloblastic leukemia, Acute myeloid leukemia	Cancer Genome Atlas Research Network et al., 2013; Huret et al., 2013; Yoshihara et al., 2015; Lee et al., 2017
***TENM2***				
t(5;21)(q35;q22.3) *TENM2/TMPRSS2*	RNA (TCGA)	Out-of-frame	Prostate adenocarcinoma	Lee et al., 2017
t(5;5)(p15;q35) *ANKH/TENM2*	DNA^***^		Neuroblastoma	Huret et al., 2013; Pugh et al., 2013
t(5;5)(q33.3;q35) *HAVCR1/TENM2*	DNA	In-frame	Breast invasive carcinoma	Banerji et al., 2012; Lee et al., 2017
t(5;5)(q34;q35) *TBC1D9B/TENM2*	RNA (TCGA)	In-frame	Lung adenocarcinoma	Huret et al., 2013; Yoshihara et al., 2015; Lee et al., 2017
t(5;5)(q34;q35) *WWC1/TENM2*	RNA (TCGA)	Out-of-frame	Breast adenocarcinoma, Ovarian serous adenocarcinoma	Yoshihara et al., 2015; Lee et al., 2017
***TENM4***				
t(11;11)(q14;q23) *TENM4/CADM1*	DNA^***^		Chronic lymphocytic leukemia	Huret et al., 2013; Puente et al., 2015
t(3;11)(p22;q14) *TENM4/DLEC1*	DNA	Out-of-frame	Small cell lung carcinoma	Huret et al., 2013; George et al., 2015; Lee et al., 2017
t(11;11)(q14;p11.2) *TENM4/EXT2*	RNA (TCGA)	5′UTR-5′UTR	Esophageal carcinoma	Lee et al., 2017
t(11;11)(q14;q24.1) *TENM4/GRAMD1B*	DNA, RNA		Chronic lymphocytic leukemia	Puente et al., 2015
t(11;11)(q14;q23) *TENM4/GRIK4*	RNA (TCGA)	Out-of-frame	Lung adenocarcinoma	Huret et al., 2013; Yoshihara et al., 2015; Lee et al., 2017
t(11;11)(q14;q14) *TENM4/INTS4*	RNA (TCGA)	Out-of-frame	Breast adenocarcinoma	Huret et al., 2013; Yoshihara et al., 2015; Lee et al., 2017
t(8;11)(p12;q14) *TENM4/NRG1*	RNA, protein	In-frame	Breast adenocarcinoma	Schaefer et al., 1997; Liu et al., 1999; Wang et al., 1999; Huret et al., 2013; Lee et al., 2017
t(11;11)(q13;q14) *TENM4/RBM4*	RNA (TCGA)	In-frame	Breast adenocarcinoma	Huret et al., 2013; Yoshihara et al., 2015; Lee et al., 2017
t(11;12)(q14;q21) *TENM4/TMTC2*	RNA (TCGA)	Out-of-frame	Breast adenocarcinoma	Huret et al., 2013; Yoshihara et al., 2015; Lee et al., 2017
t(11;11)(q14.1;q14) *AAMDC/TENM4*	RNA (TCGA)	Out-of-frame	Breast adenocarcinoma	Lee et al., 2017
t(11;11)(q13.3;q14) *ANO1/TENM4*	RNA (TCGA)	Out-of-frame	Breast adenocarcinoma	Lee et al., 2017
t(11;11)(q13.5;q14) c11orf30^*^/TENM4	DNA	In-frame	Breast adenocarcinoma	Huret et al., 2013; Nik-Zainal et al., 2016
t(11;11)(q14.1;q14) C11orf67^**^/TENM4	DNA		Breast invasive carcinoma	Banerji et al., 2012; Lee et al., 2017
t(11;11)(q13;q14) *C2CD3/TENM4*	RNA (TCGA)	In-frame	Breast adenocarcinoma	Huret et al., 2013; Yoshihara et al., 2015; Lee et al., 2017
t(3;11)(p22;q14) *DLEC1/TENM4*	RNA	Out-of-frame	Small cell lung carcinoma	Huret et al., 2013; Iwakawa et al., 2013
t(11;11)(q14;q14) *GAB2/TENM4*	RNA (TCGA)	CDS-5′UTR	Breast adenocarcinoma	Huret et al., 2013; Yoshihara et al., 2015; Lee et al., 2017
t(8;11)(p12;q14) *KIF13B/TENM4*	RNA (TCGA)	In-frame	Breast adenocarcinoma	Huret et al., 2013; Yoshihara et al., 2015; Lee et al., 2017
t(11;11)(q14.1;q14) *KCTD21/TENM4*	RNA (TCGA)	5′UTR-CDS	Breast adenocarcinoma	Lee et al., 2017
t(11;11)(p11;q14) *MTCH2/TENM4*	DNA	In-frame	Small cell lung carcinoma	Huret et al., 2013; George et al., 2015; Lee et al., 2017
t(11;11)(q14.1;q14) *NARS2/TENM4*	DNA	In-frame	Breast adenocarcinoma	Nik-Zainal et al., 2016
t(11;11)(q13;q14) *POLD3/TENM4*	RNA (TCGA)	5′UTR-5′UTR	Lung adenocarcinoma	Huret et al., 2013; Yoshihara et al., 2015; Lee et al., 2017
t(6;11)(q21;q14) *PREP/TENM4*	DNA	In-frame	Breast adenocarcinoma	Nik-Zainal et al., 2016
t(11;11)(q14;q14) *RSF1/TENM4*	RNA (TCGA)	In-frame	Breast adenocarcinoma	Huret et al., 2013; Yoshihara et al., 2015; Lee et al., 2017
t(11;11)(q13;q14) *SHANK2/TENM4*	DNA	In-frame	Breast adenocarcinoma	Nik-Zainal et al., 2016

* *c11orf30 current consensus gene name is EMSY*;

** *c11orf67 current consensus gene name is AAMDC*;

*** *present in DNA only, no expression of fusion transcript. Only rearrangements with information on tumor origin were included*.

### Mechanistic Base For Chromosomal Alterations

Under the assumption that all Teneurin genes had comparable sequence coverage in the above genome-wide approaches, these data would suggest preferential chromosome breakage at the *TENM4* locus. The presence of common or rare fragile sites, as defined by their susceptibility to form gaps and breaks in metaphase chromosomes exposed to replicative stress, has been associated with structural DNA variation, including chromosomal rearrangements, and genomic instability in cancer (Glover et al., 2017). No explicit fragile sites encompassing Teneurin genes have been described in the literature, which is consistent with the lack of findings when we performed Teneurin gene-based searches of a database covering 118 reported fragile sites in human chromosomes HumCFS, http://webs.iiitd.edu.in/raghava/humcfs/, (Kumar et al., 2017). However, an alternative mechanism proposed that transcription of large genes is associated with both copy number variation (CNV) and the formation of common fragile sites in the genome, through unrepaired lesions derived from failure at the replication fork (Smith et al., 2006; Wilson et al., 2015). Further, differential isoform expression of large genes has been associated with CNVs and common fragile sites that were cell-line specific. Based on this, we might predict that rearrangement of Teneurins could occur in a cell type-specific pattern, depending on the intrinsic transcriptional activity of each Teneurin gene and/or isoform expression in a particular tissue. This seems consistent with our previous detection of Teneurin-4 and Teneurin-2 expression in ovarian tumors and breast cancer cell lines (Graumann et al., 2017), as both genes were preferentially targeted by rearrangements in breast tumors and in one ovarian cancer sample (Table 1). Conversely, structural alterations encompassing other Teneurins might be expected in tumors less represented in current databases, such as those affecting the nervous system where Teneurin-3 expression could be more prevalent. In fact, structural alterations of *TENM3*, including one case of homozygous inactivation, occurred in 5/87 pediatric neuroblastomas and were thus considered recurrent events (Molenaar et al., 2012). The same study detected an interchromosomal rearrangement involving *CSMD2* (CUB and Sushi Multiple Domains 2) and *TENM3*, and one case of massive *TENM2* rearrangement caused by chromothripsis, a catastrophic event leading to focal chromosome shredding. Expression of a hybrid transcript encompassing *XRCC3* (X-Ray Repair Cross Complementing 3) and *TENM4* sequences was detected in a further analysis of neuroblastoma (Boeva et al., 2013). Chromothripsis associated to massive rearrangements of genes between chromosomes 3 and 11, including *TENM4*, was also described in two small cell lung cancers (SCLCs) (George et al., 2015). Together, this evidence suggests that Teneurin genes are targets of chromosomal rearrangements in multiple solid tumors. This is consistent with the proposed fragility of large transcribed genes, and the observed tissue-specificity of Teneurin rearrangements might possibly relate to their normal, intrinsic expression patterns.

Assuming the model that predicts an overlap of common fragile sites and CNVs in large genes (Wilson et al., 2015) is pertinent, additional signs of genetic instability at Teneurin gene loci should be expected in human tumors. Among these, compelling evidence suggests that oncogenic viruses recurrently integrate at genome fragile sites throughout different cancer types (Feitelson and Lee, 2007; Dall et al., 2008). Indeed, disruption of the *TENM2* gene through insertion of hepatitis B virus (HBV) DNA was reported in a liver sample affected by chronic hepatitis (Minami et al., 2005). As this condition can precede hepatic cancer, the presence of early genetic alterations might be considered as initiating events implicated in tumorigenesis. This notion is supported by an additional finding of HBV integration upstream of *TENM2* in a hepatocellular carcinoma and its corresponding normal adjacent tissue, which might share common premalignant genetic changes (Jiang et al., 2012). HBV integration in proximity to *TENM1* was detected in a fourth adjacent normal specimen, while intragenic insertion at the *TENM1* locus occurred in another tumor. HBV integration in hepatocellular carcinoma was also found to target *TENM4* (Zhao et al., 2016). In addition to HBV, the 4q35.1 locus encompassing part of the *TENM3* gene was identified among a list of recurrent integration sites for human papilloma virus (HPV) DNA in cervix cancer cells (Jang et al., 2014). Although this study did not find fragile sites within *TENM3*, integration occurred in close proximity to a DNA region interacting with the chromatin-binding BRD4 (Bromodomain Containing 4) and viral E2 proteins. A genome-wide assessment of such sites revealed that they are frequently affected by deletions and that they act to nucleate viral replication foci, whereby viral integration can occur. This mechanism, involving the generation of deletions, could also overlap with a predicted presence of CNVs in the form of deletions/duplications, described as a third hallmark of large gene fragility (Wilson et al., 2015). Accordingly, *TENM3* was classified as a large gene recurrently affected by deletions in low grade glioma in a study that analyzed genomic data derived from 30 tumor types (Glover et al., 2017). Nearby focal deletions at 4q34.3 were also recurrent in ovarian, endometrial and adrenocortical carcinoma. *TENM3* instability in the form of DNA duplication has also been reported by two independent neuroblastoma studies (Molenaar et al., 2012; Pugh et al., 2013). Importantly, a statistical assessment of gene size distributions suggested that *TENM3* structural alterations did not accumulate solely based on gene length, but were the result of active selection during tumorigenesis (Molenaar et al., 2012).

**Figure 1:**
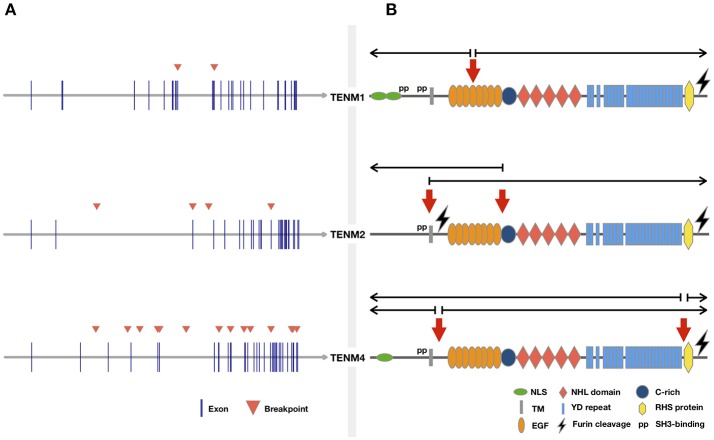
Cancer related translocations involving Teneurin genes. Chromosomal rearrangements were retrieved from the “Atlas of Genetics and Cytogenetics in Oncology and Hematology” and ChimerDB 3.0 by gene searches for each Teneurin. Rearrangements without information on tumor origin were not included. **(A)** Genomic location of breakpoints from Table 1 are indicated by arrowheads. **(B)** Predicted location of breakpoints at protein level. Domains are based on structure proposed previously (Tucker et al., 2012). The approximate breakpoint location was estimated from the exon-to-domain correspondence shown in the ENSEMBL v.93 browser. Half open arrows over the breakpoint indicate the predicted portion of the protein retained in the resulting translocation product.

Considering the previous evidence, there appears to be sufficient tumor data to document Teneurin genes as recurrent sites of targeted disruption by chromosome rearrangements, through mechanisms that include translocations, CNVs, chromothripsis, and viral genome integration. Teneurins could thus fulfill criteria to be classified as large transcribed units prone to genetic instability. Additional structural determinants related to this fragility should be examined in more detail in future studies. These include sequence-based DNA motifs and epigenetic parameters such as chromosome organization (Debatisse et al., 2012; Canela et al., 2017). Further, a meta-analysis of virus integration sites confirmed the preference of HPV and HBV for transcriptionally active regions in accessible chromatin, and revealed a consistent mark of DNA methylation and specific histone modifications at these sites (Doolittle-Hall et al., 2015). We previously predicted the presence of several CpG-rich islands at the *TENM2* and *TENM4* gene regions (Graumann et al., 2017). Although we found no evidence of methylation-based transcriptional regulation of Teneurins in tumor cells, DNA hypomethylation might influence susceptibility to viral integration and translocation events by rendering DNA more accessible, as shown for some lymphoid malignancies (Cui et al., 2013; Lu et al., 2015). Finally, the occurrence of prevalent tandem rearrangements, defined by the involvement of genes that reside on the same strand and chromosome, has been related to an increased length of introns surrounding the fusion break points of both partner genes (Greger et al., 2014). This might explain the predominance of intrachromosomal rearrangements (22/32, 69%) observed for Teneurin genes, that harbor conserved and particularly large introns between the first predicted exon sequences (Minet and Chiquet-Ehrismann, 2000) (Figure 1A). Of 21 break points included in Table 1, 12 (57%) occurred within intron sequences.

### Are Chromosomal Alterations Functionally Relevant?

RNA-level expression data was available for most Teneurin gene rearrangements reported in tumors, but concomitant protein expression was not assessed. A potential functional contribution to tumor formation can thus only be inferred. To our knowledge, the only exception relates to γ-heregulin, a secreted TENM4/NRG1 fusion protein that displayed growth-promoting biological activity (Schaefer et al., 1997). Consistent with the finding that most Teneurin translocations were unique and involved distinct fusion partners (Table 1), γ-heregulin failed to be detected in additional breast cancer cell lines and tumor specimens (Wang et al., 1999; Sánchez-Valdivieso et al., 2002). However, the affected break region maps to a recurrent rearrangement site involving chromosomes 8 and 11 in breast and pancreatic tumors (Adélaïde et al., 2000). Further, in a subset of early-onset pancreas cancers, *NRG1* rearrangement correlated with wild-type *KRAS* and susceptibility to pharmacologic ERBB inhibition (Heining et al., 2018). This matches the biologic behavior of MDA-MB-175 cells expressing γ-heregulin, which do not carry *KRAS* mutations (Hollestelle et al., 2007) and are sensitive to pertuzumab-mediated inhibition of HER3 (ERBB3) signaling in a mouse xenograft model (Lee-Hoeflich et al., 2008). Together, these data suggest that retention of *TENM4* N-terminal sequences in γ-heregulin, which include the entire intracellular and transmembrane domains (Schaefer et al., 1997), generates a functional gene product with oncogenic activity comparable to that of other *NRG1* fusion partners. Conservation of the 5′-N-terminal TENM4 domains is recurrent to other translocations (Figure 1B).

For chromosomal alterations that lacked functional assessment, some general points should be considered. First, different outcomes would be expected for gene disrupting rearrangements, as opposed to those generating chimeric proteins through gene fusion. In the former category, gene disruption through recurrent deletion was only described for *TENM3* in gliomas (Glover et al., 2017), and homozygous loss of *TENM3* was also found in an embryonal rhabdomyosarcoma associated with high risk clinical parameters (Walther et al., 2016). No comparable deletion hotspots have been reported at the *TENM1, TENM2*, and *TENM4* loci (Hazan et al., 2016). However, about one third of chromosomal translocations (Table 1) resulted in out-of-frame gene fusions. Interruption of the reading frame is typically associated with a premature translation halt leading to degradation of faulty products, through mechanisms such as nonsense-mediated mRNA decay (He and Jacobson, 2015). Rarely, translation of functional truncated proteins from such rearrangements has been documented (Mertens et al., 2015; Rodriguez-Perales et al., 2016). As Teneurin genes were in a 5′ position in 5/9 out-of-frame rearrangements, expression of a truncated form is unlikely, but theoretically possible. A third mechanism associated to gene disruption occurred through viral insertional mutagenesis, though the effect on Teneurin gene expression was not assessed (Minami et al., 2005; Jiang et al., 2012; Jang et al., 2014; Zhao et al., 2016). In fact, HBV appears to recurrently integrate at Teneurin gene loci. Integration of this virus was found to preferentially target cancer genes and actively transcribed genome sites, consistent with a potential gene disruptive outcome (Doolittle-Hall et al., 2015). Finally, Teneurin genes suffered massive, disruptive rearrangements through chromothripsis (Molenaar et al., 2012; George et al., 2015). When considered together, these mechanisms suggest that a subset of structural alterations might lead to inactivation of Teneurin gene expression in some tumors. The heterozygous loss of function, potentially leading to a haploinsufficient phenotype, could be consistent with a tumor suppressive role in such cases.

In contrast, a different outcome might be expected for rearrangements generating hybrid transcripts. As shown in Table 1, expression of fusion-derived RNA was asserted for 20/31 (64.5%) translocations. Of all predicted fusion products, 13/32 (40.6%) were in-frame and at least 2 placed novel 5′-UTR sequences ahead of a Teneurin gene, potentially enabling chimeric protein expression or transcriptional control through a foreign regulatory region, respectively. Interestingly, 8/9 (89%) out-of-frame rearrangements were also detected at the RNA level, supporting their transcriptional expression. However, such forms might not be active in terms of protein production, as suggested by the low translation index of diverse out-of-frame chimeras transcribed in breast cancer cells (Inaki et al., 2011), and as discussed above. With regard to translocations substituting 5′-UTR regulatory sequences, placement of a strong *IGH* promoter upstream of *TENM2* was associated with at least 3-fold higher Teneurin-2 transcript levels in MALT lymphomas, as compared to tumors not bearing the translocation (Vinatzer et al., 2008). This finding is highly reminiscent of *IGH* promoter-driven overexpression of *MYC, CCND1* (Cyclin D1), *BCL2*, and *BCL6* proto-oncogenes in B-cell malignancies (Zheng, 2013). Importantly, gene translocations have been recognized as early clonal events in hematologic malignancies, and have been associated with causative roles in oncogenic transformation (Mitelman et al., 2007). Since the *IGH/TENM2* rearrangement was recurrent in 3 tumors, its phenotypic impact should be investigated. The consequence of other 5′-UTR substitutions is less evident. Leukemias carrying the *KSR1/TENM1* rearrangement showed a hybrid transcript ratio of 1.0 (Yoshihara et al., 2015), indicating lack of endogenous Teneurin-1 expression with sequencing reads solely derived from the fusion transcript. Since basal expression of *KSR1* (Kinase Suppressor of Ras 1) was reported in HL60 acute promyelocytic leukemia cells (Wang et al., 2006), a transcriptionally active *KSR1* promoter might be expected in leukemia and drive *de novo* expression of *TENM1*, with potential functional consequences. No equivalent expression data was specified for the *KCTD21/TENM4* translocation detected in breast adenocarcinoma. In *ERBB2*-amplified breast cancer, expression of *KCTD21* (Potassium Channel Tetramerization Domain Containing 21) was related to genomic copy number alterations (Sircoulomb et al., 2010). The corresponding 5′-UTR could thus be active in a subset of breast cancers and drive *TENM4* expression in the above case. Although validation is missing, this evidence suggests that Teneurin (over)expression could be driven through foreign upstream regulatory sequences in some tumors, suggestive of an oncogenic rather than tumor suppressive role for this mechanism.

Third, almost 40% of translocations (Table 1) were predicted to generate in-frame gene fusions, which expressed a corresponding hybrid transcript in all cases where RNA was assessed. This frequency agrees with findings derived from 13 cancer types, where 36% of gene fusions were in-frame (Yoshihara et al., 2015). In the current set, 10/13 in-frame rearrangements occurred in breast tumors. The presence of chromosomal rearrangements in solid tumors has increasingly been reported in recent years and is a matter of current investigations (Mertens et al., 2015). Interestingly, mutation of relevant cancer genes was significantly reduced in breast cancers and other tumors carrying in-frame fusion transcripts, suggesting the latter could function as driver events (Yoshihara et al., 2015). In light of missing functional data to support an analogous role for Teneurin rearrangements, an indirect appraisal might be gained through the analysis of the corresponding fusion partners. Not surprisingly, most of these genes (Table 1) have been implicated in the context of malignant diseases. Among the in-frame fusion partners, *EMSY* (c11orf30), a BRCA2-interacting transcriptional repressor involved in DNA repair, is frequently amplified in breast, ovarian and lung cancers, where it was proposed to exert oncogenic functions (Hughes-Davies et al., 2003; Baykara et al., 2015). For the hepatitis virus A receptor gene, *HVCR1*, overexpression has been well-documented in clear cell renal cancer and shown to induce growth and angiogenesis-promoting factors (Cuadros et al., 2014). *KIF13B*, a member of the kinesin gene family, is involved in trafficking of the Vascular Endothelial Receptor 2 (*VEGFR2*), and disrupting this interaction has demonstrated anti-angiogenic potential in a lung tumor xenograft model (Yamada et al., 2017). Of note, a *KIF13B/NRG1* fusion was identified in a liver metastasis and associated with tumor progression (Xia et al., 2017). Both *KIF13B* and *NRG1*genes can thus act as Teneurin fusion partners. For all remaining in-frame fusion partners with available cancer-related studies, equivalent results supported their oncogenic roles through over-expression or gene amplification. Only two genes showed opposite behaviors consistent with tumor suppressive functions. Hence, decreased expression of the mitochondrial transporter *MTCH2*, a pro-apoptotic gene, was associated with enhanced invasiveness and tumor progression in various tumor types (Yu et al., 2008; Arigoni et al., 2013). Similarly, *RBM4* was shown to suppress tumor progression and to counter a colorectal metastatic cascade through modulation of alternative splicing (Wang et al., 2014; Lin et al., 2018), and its expression was decreased in various tumor types. For *CDCD3* (Desmin), only one cancer related publication could be retrieved that reported a *MS4D7/CDCD3* translocation with unknown function in oropharyngeal cancer (Wang et al., 2013). For out-of-frame fusion partners, evidence for both oncogenic (*ANO1, TMPRSS2*) (Ko et al., 2015; Wang et al., 2017) and tumor suppressive roles (*DLEC1, WWC1*) (Knight et al., 2018; Li et al., 2018b) could be identified. Of note, all Teneurin fusion partner genes in the current set have been involved in additional translocations (documented at the “Atlas of Genetics and Cytogenetics in Oncology and Hematology,” http://atlasgeneticsoncology.org/), albeit at varying frequencies. This suggests that, besides their documented roles in cancer, these genes are frequent targets of structural rearrangements in tumors with probable functional consequences. Thus, Teneurin rearrangements are not incidental and preferentially involve genes related to cancer.

Finally, additional evidence pertinent to tumor gene rearrangements should briefly be considered. Of particular relevance to Teneurins, large transcriptional units prone to breakage in cancer cells show a frequent involvement in neurological development (Smith et al., 2006). This would agree with alterations in Teneurins and other genes that regulate neuritogenesis, which were associated with an aggressive phenotype in neuroblastoma (Molenaar et al., 2012). A role in neurological development and/or disorders is also known for some Teneurin fusion partners (e.g., *NRG1, NARS2, SHANK2, GRIK4, TMTC2*, and *KIF13B*), as revealed by gene-related information available at Gene Cards (https://www.genecards.org/). Further, large genes often display a high degree of evolutionary conservation, which extends to intronic sequences and fragile sites acting as preferential targets for chromosomal breakage (Greger et al., 2014; Glover et al., 2017). Not surprisingly, genome caretaker, and tumor suppressor functions have been postulated for frequently altered large genes, whose loss might provide a selective advantage to affected cells (Hazan et al., 2016; Karras et al., 2016). Gene conservation is thus considered a possible hallmark of essential cellular functions, which include the DNA damage and stress induced responses (Smith et al., 2006; Hazan et al., 2016). These features might well fit the highly conserved Teneurin genes (Tucker et al., 2012). In the particular case of Teneurin-4, gene expression further responds to conditions of endoplasmic reticulum stress, although a direct biological consequence has not been established (Wang et al., 1998). Finally, high-throughput RNA sequencing revealed that chimeric transcripts resulting from chromosomal rearrangements are expressed in a prominently tissue specific manner (Frenkel-Morgenstern et al., 2012). It was observed that chimeras often retained signal peptides and transmembrane regions, which might redirect potential hybrid proteins to unusual subcellular compartments. As shown in Figure 1B, all rearrangements retaining Teneurin intracellular domains preserved the transmembrane region, providing potential membrane anchorage to the 3′ fusion partner. In summary, distinct types of genomic rearrangements can lead to a range of potential outcomes for a same gene, including both oncogenic and tumor suppressive contributions to tumor development (De Braekeleer et al., 2012). Based on current evidence, a similar scenario seems highly probable for Teneurin genes.

## Transcriptomic Evidence of Dysregulated Teneurin Gene Expression

Depending on the tumor context, cancer genes can be targeted by different activating and/or inactivating mechanisms, both genetic and epigenetic. Consistent with this, altered expression of Teneurins has been reported for various tumors, in addition to structural alterations discussed above. Previously, we had reviewed transcriptomic data and reported on evidence of decreased Teneurin expression in cancers of the liver, esophagus, and kidney, while increased expression was found for brain tumors and lymphomas (Ziegler et al., 2012). With exception of increased Teneurin-2 levels related to the *IGH/TENM2* translocation, accompanying data to explain the biological basis of these changes was not available. However, gene disruption through HBV insertion might be associated to reduced Teneurin-2 levels in hepatocellular carcinoma and was suggested to occur early in tumor development (Minami et al., 2005). For Teneurin-2, further data supported an early time point for expression changes in carcinogenesis of the breast (Lee et al., 2007), while in cervical cancer, RNA levels decreased in advanced stages with nodal compromise or the presence of metastasis (Noordhuis et al., 2011). Based on this data, a first appraisal suggested that both Teneurin up- and downregulation could occur in tumors, with a potential involvement in cancer initiating events as well as in tumor progression.

### Is Teneurin Gene Expression Regulated by Epigenetic Mechanisms?

Since epigenetic modifications are a frequent cause of aberrant gene expression in tumors, we had previously analyzed the effect of a demethylating agent (5-azacytidine) on the expression of Teneurin-2 and Teneurin-4 in cancer cells (Ziegler et al., 2012; Graumann et al., 2017). Although the presence of CpG-rich regions within several Teneurin genes was predicted by us and others (Beckmann et al., 2011), we found no evidence for an effect of DNA demethylation on Teneurin-2 and Teneurin-4 expression in breast and ovarian cancer cell lines. However, analysis of the *TENM3* promoter region revealed increased methylation in early breast lesions as compared to normal breast tissue (Tommasi et al., 2009), and in immortalized keratinocytes, Teneurin-4 gene expression could be downregulated by overexpression of DNMT3B, an enzyme involved in *de novo* methylation of DNA (Peralta-Arrieta et al., 2017). This evidence would favor a DNA methylation-based downregulation of Teneurin expression in a manner analogous to that of other tumor suppressor genes, perhaps in a tissue-specific manner. A main drawback of these studies, however, is that concurrent analysis of DNA methylation and transcript expression were not performed, precluding conclusions on a causal relationship between both processes. To date, a single report accomplished such assessment and could demonstrate that, in a subset of glioblastoma cells, methylation of *TENM1* upstream sequences was indeed inversely correlated with transcript expression (Talamillo et al., 2017). For Teneurin-1, a further role in modulating the epigenetic landscape has been proposed based on the interaction of its cleaved intracellular domain with MBD1, a nuclear CpG-binding transcriptional repressor (Nunes et al., 2005). MBD1 participates in mechanisms that regulate heterochromatin formation, and in tumors, it has been associated with silencing of tumor suppressor genes, promotion of oncogenic attributes, and a reduced response to cisplatin and radiation (Li et al., 2015; p. 1). Interestingly, MBD1 is highly expressed in neural stem cells and its defects can impair neuron differentiation (Li et al., 2015; p. 1), underscoring its close functional link to Teneurins in non-tumor tissues. These data suggest that Teneurin-1 might be part of an epigenetic regulatory circuit, both as a modulator of gene expression and as a target of methylation-based gene silencing. However, it should be kept in mind that, owing to its cytogenetic localization, Teneurin-1 might be subjected to distinct epigenetic control mechanisms related to gender-dependent X-chromosome inactivation. Whether other Teneurin genes are regulated by equivalent methylation-based mechanisms remains to be demonstrated. As an example, differential methylation of *TENM2, TENM3*, and *TENM4* was reported in a genome-wide analysis of neuroblastoma, but no findings for *TENM1* were registered (Gómez et al., 2015). Further, methylation occurred outside of CpG islands most commonly associated with methylation-based transcriptional control, and methylation changes were not correlated with altered Teneurin gene expression. This data suggests that, although *TENM1, TENM2*, and *TENM3* genes might show tumor-related differences in methylation patterns, *per se* this does not imply a concomitant change in gene expression. These aspects demand comprehensive analyses that remain to be addressed. At present, epigenetic control has been sufficiently documented only for *TENM1*.

### Uncovering Teneurin Gene Regulatory Pathways in Tumors: The NOTCH Connection

As discussed above, DNA methylation seems not sufficient to explain tumor-related changes in Teneurin gene expression. A reasonable alternative should consider Teneurins as downstream targets of cancer-specific cellular signaling, which might impact at the level of gene expression, proteolytic protein processing, or the regulation of specific domain activity by posttranslational modifications, among others. This would be consistent with the fact that Teneurin translocation partners were enriched in cancer-related genes, which delineates a well-described oncogenic mechanism. With regard to signaling pathways, vast structural and functional parallels between Teneurins and Notch proteins have recurrently been noted, which include their analogous transmembrane localization, their capability to dimerize upon ligand interaction, the presence of multiple epidermal growth factor-like (EGF) repeats in their extracellular domains, and their processing into multiple domains through proteolytic cleavage (Tucker and Chiquet-Ehrismann, 2006; Schöler et al., 2015; Vysokov et al., 2016). These associations were recently shown to reach deeper, as overexpression of the NOTCH1 intracellular domain, which functions in a nuclear transcription activator complex, revealed that *TENM4* is a NOTCH-responsive gene (George et al., 2015). The authors proposed that NOTCH signaling acts as a key regulator of neuroendocrine differentiation in small cell lung cancer. This seems consistent with a potential effector role of Teneurins, considering their essential participation in neural development (Tucker et al., 2007). *TENM4* responsiveness to NOTCH signaling was also noted in muscle satellite cells, where both were required for maintenance of cell quiescence and inhibition of myogenic differentiation (Bröhl et al., 2012; Ishii et al., 2015). In small cell lung cancer, NOTCH signaling was ascribed a tumor suppressive role, and as a NOTCH-regulated gene, an equivalent function might be expected for *TENM4*. Notably, the similarities between both gene families go well-beyond the observations outlined above, and could define a highly probable setting governing Teneurins' involvement in cancer. As recently reviewed in great detail (Nowell and Radtke, 2017), the contribution of NOTCH-mediated signaling is particularly dependent on the cellular and tumor context, with both oncogenic and tumor suppressive outcomes. Oncogenic activation can occur through chromosome translocations or activating NOTCH mutations in leukemia, while inactivating mutations occur in tumors where it exerts a tumor suppressive role, such as in small cell lung cancer (George et al., 2015). Further, *NOTCH4* is a target for mouse mammary tumor virus (MMTV) integration in mice, and NOTCH gene expression can be altered by some HPV viral proteins, unveiling a role for viral-dependent mechanisms that shows remarkable similarities with Teneurins. The phenotypic contribution of NOTCH dysregulation could relate to its ability to negatively and positively regulate differentiation and stem cell fate, in analogy to the role of Teneurins in modulating cellular differentiation in different cell types (Suzuki et al., 2012, 2014a; Ishii et al., 2015; Tews et al., 2017). Not surprisingly, expression of NOTCH family genes has been associated with clinical parameters and patient outcome in cancer. Based on the vast implications of NOTCH signaling in malignant diseases, this pathway is a current target for development of directed therapeutic interventions.

### Teneurin Expression Is Associated With Biological and Clinical Parameters

Assuming that Teneurins are active players in tumorigenesis through mechanisms analogous to NOTCH, similar biological and clinical findings would be expected for this gene family. Not surprisingly, Teneurin expression has been related to tumor behavior and patient survival in several cancer types. In invasive and aggressive-invasive prolactin pituitary tumors, upregulation of Teneurin-1 mRNA was associated with tumor progression (Zhang et al., 2014), and a similar observation was made for papillary thyroid cancer (Cheng et al., 2016). In the latter case, Teneurin-1 expression was further associated with extra-thyroidal invasion, an advanced disease stage, the risk of recurrence, and the presence of *BRAF* V600E, an actionable mutation with a known prognostic significance in this cancer. For Teneurin-3, decreased levels predicted a worse survival in neuroblastoma patients (Molenaar et al., 2012), while expression was upregulated in breast tissue of nulliparous women, known to be at higher risk of developing breast cancer (Balogh et al., 2006). Interestingly, differentiation of breast tissue is not fully accomplished in these women and maintains a high proportion of stem cells, suggesting that upregulation of Teneurin-3 might relate to an altered differentiation process, as discussed above. In the same line, we found a decrease in Teneurin-4 expression in high grade serous ovarian tumors that undergo dedifferentiation, and reduced Teneurin-2 levels predicted a poor survival in these patients (Graumann et al., 2017). To retrieve additional prognostic associations, we performed a gene-based query at the Human Protein Atlas (https://www.proteinatlas.org/pathology), which provided statistical correlations of patient survival based on quantitative analysis of transcriptomic data. Table 2 summarizes Log-rank *p*-values based on either optimal or median separation of low and high-expressing tumor groups, providing evidence for significant prognostic implications of Teneurin expression in a range of different cancers. Interestingly, all four Teneurins were associated with patient survival in cancers of the endometrium, kidney and stomach, and with the exception of Teneurin-3 in renal cancer, survival was improved for patients with low Teneurin levels, possibly hinting to a common underlying mechanism. These findings raise the question whether all Teneurins were concomitantly expressed in these tumors. At least in some cell lines, we could demonstrate a simultaneous expression of Teneurin-2 and Teneurin-4 (Graumann et al., 2017), and coexpression of different Teneurins was also noted in one neuroblastoma cell line (Suzuki et al., 2014b), although the functional implications remain unknown. In contrast, other tumors showed an apparent gene-specific association, such as melanoma and colorectal cancer (Teneurin-2 only) and cervical cancer (Teneurin-3 only) (Table 2). About one third of cancers showed a better survival upon increased Teneurin expression, suggesting a distinct biological behavior for this group. Hence, results based on transcriptomic analysis of large TCGA patient cohorts strongly support an involvement of Teneurins in tumor biology, evidenced through their prognostic association with patient survival and tumor differentiation. It is highly probable that, as additional cancer types will be analyzed in sufficiently large numbers, new findings will emerge in a near future. Based on current data, it is conceivable that Teneurins' role in tumorigenesis could relate to their ability to modulate cell differentiation, in addition to other processes discussed below.

**Table 2:** Significant associations between Teneurin transcript levels and patient survival.

**Gene**	**Cancer type**	**Optimal separation *p*-value**	**Median separation *p*-value**	Better survival outcome^*^
***TENM1***	Breast	1.71E-03	7.89E-03	High
	Endometrial	8.68E-04	6.37E-03	Low
	Glioma	7.73E-03	1.86E-01	Low
	Head and neck	3.51E-02	4.89E-02	High
	Lung	2.68E-04	6.36E-03	High
	Pancreatic	4.49E-03	5.91E-02	High
	Renal	2.71E-02	8.98E-01	Low
	Stomach	4.41E-02	7.08E-01	Low
	Thyroid	3.25E-03	4.46E-01	High
	Urothelial	4.44E-02	1.92E-01	Low
***TENM2***	Colorectal	2.94E-02	2.85E-01	High
	Endometrial	9.16E-03	4.32E-02	Low
	Glioma	6.28E-03	5.19E-02	Low
	Head and neck	3.81E-03	6.85E-02	Low
	Melanoma	4.62E-02	4.62E-02	Low
	Ovarian	2.10E-02	2.40E-01	High
	Pancreatic	3.68E-03	2.37E-01	High
	Prostate	4.34E-02	2.83E-01	High
	Renal	4.69E-05	1.04E-02	Low
	Stomach	3.47E-02	5.78E-01	Low
	Thyroid	5.16E-04	1.72E-01	Low
	Urothelial	7.52E-04	2.57E-03	Low
***TENM3***	Cervical	3.01E-02	5.25E-01	High
	Endometrial	4.43E-02	4.99E-01	Low
	Glioma	1.26E-02	3.91E-02	Low
	Lung	8.68E-03	7.39E-02	Low
	Ovarian	2.31E-02	6.79E-02	Low
	Pancreatic	4.85E-02	3.81E-01	High
	Renal	4.10E-02	3.54E-01	High
	Stomach	1.47E-03	5.67E-02	Low
	Thyroid	1.07E-02	7.42E-02	Low
	Urothelial	5.25E-04	6.37E-02	Low
***TENM4***	Endometrial	2.96E-03	7.21E-01	Low
	Liver	1.18E-02	4.87E-02	Low
	Renal	6.51E-03	7.17E-02	Low
	Stomach	5.40E-03	4.71E-02	Low

* *“High” and “Low” refer to Teneurin transcript expression level groups according to applied threshold, defined by either Optimal separation or Median expression levels*.

### Cancer Pathways: Interaction Points of Teneurins and WNT Signaling

Considering the above associations with clinicopathological parameters, it seems reasonable that cancer-related biological mechanisms might regulate—or be regulated by—Teneurins. Besides the dazing relation between Teneurins and NOTCH, several studies are consistent with this prediction. For instance, in node-positive, poor prognosis cervical cancers, Teneurin-2 levels were increased together with CTNND1 (Noordhuis et al., 2011), a member of the catenin gene family that stabilizes E-Cadherin at epithelial adherens junctions and mediates non-canonical WNT signaling (Schackmann et al., 2013). Upon E-Cadherin loss, CTNND1 mislocalizes to the cytoplasm and aberrantly regulates Rho-mediated signals, leading to the induction of a migratory and invasive phenotype through epithelial-mesenchymal transition (EMT). Interactions between Teneurins and WNT signaling are also relevant to normal embryonal development. Hence, Teneurin-4 was a main binding partner of Olfactomedin-1 (OLFM1), a secreted glycoprotein involved in regulation of proliferation and differentiation of neural progenitor cells in the brain, and proposed to regulate small GTPase RhoA activity and WNT signaling (Nakaya et al., 2013). In avian limb development, a potential interaction between the WNT7A ligand and Teneurin-1 or Teneurin-3 was suggested based on their expression in common cellular compartments (Bagutti et al., 2003). A similar association might be deduced from the concomitant decrease in WNT7A and Teneurin-4 expression observed in neurons of schizophrenia patients obtained by *in vitro* differentiation (Brennand et al., 2011). Further, defects in WNT7A impair female genital tract development and have been associated with Müllerian duct anomalies in mice (Choussein et al., 2017), while in *C. elegans*, disruption of Ten-1 impairs development of somatic and germline gonadal cells (Drabikowski et al., 2005). These data suggest that during development, Teneurin expression might respond to WNT7A-mediated signals in some common compartments, thereby altering biological processes such as cell adhesion and migration. However, these associations do not always hold and especially in tumors, the role of WNT7A is more complex as it can exert opposite oncogenic as well as tumor suppressive functions (Stewart, 2014; Huang et al., 2018). In fact, Teneurin-1 and EMX2 levels were highly increased and HOXA10 levels were reduced in women with Müllerian defects leading to a partially separate uterus (Zhu et al., 2016). Activity of the homeobox transcription factor HOXA10, a direct negative regulator of EMX2 gene expression, is dependent on an intact WNT7A function (Miller and Sassoon, 1998). In turn, EMX2 is a direct activator of *TENM1* transcription (Beckmann et al., 2011). This would define an opposite signaling cascade where WNT7A positively regulates HOXA10 function, leading to repression of EMX2 and its downstream targets, including Teneurin-1. Since EMX2 has an antiproliferative function in endometrial cells (Taylor and Fei, 2005), its elevated expression seems consistent with the impaired cell growth associated with Müllerian duct anomalies, where Teneurin-1 could act as growth restricting downstream effector. Equally, Kaplan-Meier estimates available at The Human Protein Atlas (https://www.proteinatlas.org/pathology), register improved survivals for patients with endometrial cancer expressing high EMX2 (log-rank test *P* = 0.0054) and low WNT7A (*P* = 0.00003) levels, indicating an advantage of persistent antiproliferative signals. However, the same does not hold for Teneurin-1, whose low expression was associated with improved survival in these patients (Table 2). This result further contrasts findings in *C. elegans*, where germline tumors resulted upon deletion-mediated loss of expression of the Ten-1 ortholog (Drabikowski et al., 2005). Thus, prognostic conclusions can not necessarily be extrapolated from predicted pathway interactions and must consider additional factors, as exemplified by the finding of both positive and inverse associations between Teneurins and WNT7A in different cellular contexts.

Recently, a deeper insight has been provided into molecular mechanisms underlying the role of Teneurin-1 in cancer. The authors showed that in glioblastoma cells, loss of Teneurin-1 expression through chromosomal deletion or epigenetic silencing was associated with resistance to serum-induced differentiation (Talamillo et al., 2017). Although exogenous Teneurin-1 expression restored a differentiated phenotype, it provided cells with an enhanced migratory and invasive potential, suggesting a fine equilibrium between Teneurin-1-mediated regulation of differentiation fate and migratory capacity. Further, increased Teneurin-1 levels were predictive of a poor outcome in glioblastoma patients and xenograft models, consistent with transcript-based survival estimates for glioma patients (Table 2). Strikingly, the cleaved intracellular (ICD) but not the extracellular Teneurin-1 domain was capable of eliciting the migratory and invasive properties, as well as a rearrangement of the actin cytoskeleton, the expression of mesenchymal markers, and an increased resistance to toxicity mediated by the alkylating agent temozolomide. In functional terms, the Teneurin-1 ICD acted in the nucleus through direct interaction with the MYC oncoprotein, inducing transcriptional activation of the small GTPase *RHOA* gene. These findings place Teneurin-1 as an executor of MYC-RHOA-induced responses, which are associated with oncogenic signaling through WNT pathways in glioblastoma, although additional components of this pathway were not analyzed (Lee et al., 2016). This study strengthens the above evidence of a functional link between Teneurins and WNT signaling, and points to the relevance of proteolytic processing in the generation of Teneurin domains with distinct functional attributes. Similar findings had been described earlier for the Teneurin-2 and Teneurin-3 ICDs, which were implicated as negative regulators of ZIC1 function and ZIC2 expression, respectively (Bagutti et al., 2003; Glendining et al., 2017). The ZIC transcription factors appear to accomplish mainly tumor suppressive roles, but overexpression occurs in some cancers, suggesting an alternative oncogenic function (Houtmeyers et al., 2018). At least for the Teneurin-3 ICD, a direct interaction with ZIC2 could be demonstrated *in vitro* (Glendining et al., 2017). Interestingly, the activity of ZIC transcription factors can be inhibited in *C. elegans* neural progenitors by WNT downstream effectors ß-catenin and TCF (Murgan et al., 2015), raising the possibility that Teneurin ICDs might act as part of this protein complex to modulate transcription factor activity. In addition, Teneurin-3 knock-out experiments showed that its ICD can act as a positive and negative regulator of Ephrin receptors EPHA7 and EPHB1 expression, respectively (Glendining et al., 2017). The concomitant decrease in ZIC2 and EPHB1 is consistent with the positive regulatory role that ZIC2 exerts on *EPHB1* transcription (García-Frigola et al., 2008). Ephrin receptors activate signaling cascades through RHOA, AKT and ERK, to regulate cell growth, migration, and EMT, and their expression is altered in numerous tumor types (Kou and Kandpal, 2018). Hence, modulation of Ephrin-mediated processes provides an additional explanation of Teneurins contribution to tumor development. Further, the distinct activities of cleaved Teneurin ICDs imply that their preservation in chromosomal translocation products (Figure 1B) might actively contribute to oncogenic functions, through ICD-mediated transcriptional modulation of relevant target genes (Bagutti et al., 2003). Since proteolytic release of the Teneurin-2 ICD can be promoted by homophilic interactions (Bagutti et al., 2003), it should be examined if alternative ligand-dependent mechanisms operate in tumor cells, perhaps as the result of Teneurin interactions with components of the extracellular matrix (ECM). Thus, current evidence supports a role of Teneurins as mediators or effectors of WNT signaling, which might be associated with tumor suppressive as well as oncogenic outcomes. We had previously proposed a model to describe such potential interactions (Ziegler et al., 2012).

### Teneurins and Modulation of Neuregulin-ErbB-Mediated Signaling

In cancer cells, different signaling pathways often converge at common cross-points to establish complex regulatory networks. It is thus not surprising that Teneurins might appear in different molecular contexts directed at a common functional endpoint. As noted above, a hybrid *NRG1/TENM4* fusion product (γ-heregulin) displayed growth promoting activity on different tumor cell lines (Schaefer et al., 1997). Although these experiments focused on growth stimulation associated with the secreted Neuregulin-1 ligand, it should be recalled that proteolytic cleavage of γ-heregulin would simultaneously generate a membrane anchored Teneurin-4 ICD, which might exert additional roles to support oncogenic transformation. Interestingly, NRG signaling through ERBB receptors accomplishes crucial functions in nervous system development that are highly reminiscent of Teneurins (Mei and Nave, 2014). Moreover, single nucleotide variants (SNVs) in NRG and ERBB genes have been associated with an increased risk of psychiatric conditions such as schizophrenia and bipolar disorder (Mei and Nave, 2014), analogous to findings reported for Teneurin-4 (Psychiatric GWAS Consortium Bipolar Disorder Working Group, 2011; Ivorra et al., 2014). Similarly, neurons from schizophrenia patients differentiated *in vitro* showed misexpression of various genes including *TENM4, NRG1* and *ERBB4*, suggestive of a functional connection between these proteins. A similar association might be inferred from experiments with overexpression of the Teneurin-1 ICD in glioblastoma cells, which lead to an increased transcriptional activation of melanogenesis-associated transcription factor (MITF) target genes, including *ERBB3* (Schöler et al., 2015). Teneurins might thus support this oncogenic pathway by promoting the expression of ERBB receptors to provide sufficient binding sites for NRG ligands. In line with this notion, a recent study using CRISPR-CAS9 to knock-out ZEB1, a homeobox transcription factor that mediates epithelial to mesenchymal transition (EMT) in response to TGF-ß, found that Teneurin-2 transcription is directly repressed by ZEB1 in triple-negative breast cancer cells (Maturi et al., 2018). Interestingly, ERBB4 expression increased over 5-fold upon ZEB1 knockout, with a concomitant 16-fold increase in Tenerin-2, which agrees with the association proposed for these gene families. In the case of breast cancer, it is interesting to speculate that the positive prognostic impact of Teneurin-1 overexpression (Table 2) might be associated with a simultaneous expression of ERBB receptors, which provide an actionable target for directed therapies able to positively impact on patient survival (Hynes, 2016). This interaction illustrates how additional factors can modify an expected prognostic impact predicted by molecular parameters. As a further example, ZEB1 knock-out cells showed diminished invasiveness and a delayed migratory behavior, which might predict a positive prognostic impact of Teneurin-2 reexpression. However, the opposite was true in a small group of patients with triple-negative breast cancer, where increased Teneurin-2 was associated with shorter metastasis-free survival (Maturi et al., 2018). A possible explanation might relate to the poor prognostic impact of ERBB4 expression in triple negative breast cancer patients receiving standard, non-targeted treatment regimens (Kim et al., 2016). In the former study, the therapeutic modalities were not specified and preclude assessment of this variable, which might modify the prognostic impact of Teneurins in a treatment-dependent manner (Maturi et al., 2018).

If the proposed association holds, Teneurins would be linked to additional cancer signaling pathways, as NRG1/ERBB-dependent phosphorylation triggers PI3K/AKT and MEK/ERK-mediated responses (Roskoski, 2014). This interaction should be analyzed in depth in normal and tumor cells, as it involves essential mechanisms in both contexts. To analyze if coexpression of Teneurin and ERBB genes occurs frequently in tumors, transcriptomic analyses of larger sample groups are required and should consider the therapeutic modalities received by patients as key determinants of patient outcome. The molecular role of Teneurin ICDs in mediating transcriptional activation of ERBB genes should also be assessed.

### Additional Functional Considerations

Another aspect to be considered is the well-documented interaction of Teneurins with cytoskeletal and extracellular matrix (ECM) components, which could bear potential relevance to cancer. Hence, a role for Teneurins in cell migration was recognized early in *Drosophila*, as heterophilic interaction of Ten-m with PS2 integrins was shown to promote cell spreading (Graner et al., 1998). Further, disruption of Ten-m or the actin-crosslinking protein Filamin resulted in a comparable phenotype characterized by altered cell migration and routing of motor neurons (Zheng et al., 2011), suggesting a connection between Teneurins and the actin-based cytoskeleton in cell motility. The authors could also demonstrate that Filamin and Ten-m physically interact in epidermal cells. Interestingly, actin cytoskeleton dynamics appears to be regulated by both the Teneurin-1 ICD (Talamillo et al., 2017) and the Teneurin C-terminal associated peptide (TCAP-1) (Chand et al., 2012). These constitute two structurally unrelated domains located on opposed Teneurin protein termini, derived either through proteolytic cleavage or by transcription from alternative intronic promoters. In mouse hypocampal cells, cellular effects of the secreted TCAP1 domain were mediated by interaction with a dystroglycan receptor complex. This elicited the activation of MEK and ERK-dependent signaling, leading to filamin phosphorylation and actin polymerization required for outgrowth of cellular protrusions. In contrast, the Teneurin-1 ICD acted through its proposed nuclear role in transcriptional regulation by inducing *RHOA* gene expression, with resulting activation of Rho-dependent kinase (ROCK) signaling and remodeling of the actin cytoskeleton. In this case, glioblastoma cells acquired features of increased tumor cell aggressiveness, as discussed above. No evidence is currently available to support the concomitant activity of TCAPs and Teneurin ICDs in tumors or normal tissues. TCAPs are bioactive peptides capable of eliciting intracellular signals that could be relevant to tumorigenesis (Wang et al., 2005). These issues deserve further clarification and illustrate the complex mechanisms that underlie the activity of Teneurins.

With regard to interaction of Teneurins with components of the extracellular matrix (ECM), the integrity of basal membrane structures in *C. elegans* was dependent on the interaction of Ten-1 at the surface of epidermal cells with collagen IV in the extracellular space (Topf and Chiquet-Ehrismann, 2011). Conversely, Teneurin-4 was shown to negatively regulate expression of collagens type II and X through mechanisms involving ERK-dependent signaling, which was associated with suppression of chondrogenic differentiation and preservation of a mesenchymal phenotype (Suzuki et al., 2014a). Thus, Teneurins can alter determinants of cell adhesion and migration, which are essential targets in tumor cells, by direct interaction and modulation of components of the ECM. Interestingly, a proteomic study revealed a prominent increase of membrane-associated Teneurin-1 upon activation of platelets with a collagen-related peptide (Wright et al., 2011). Platelets are important promoters of tumor development through mechanisms that include the release of proangiogenic and growth promoting factors (Plantureux et al., 2018). Further, platelets adhere to tumor cells and protect them against mechanical forces and immune surveillance in the bloodstream, providing an essential contribution to metastatic spreading. The adhesion of tumor cells to platelets is mediated by various cell surface molecules such as integrins, P-selectin, and podoplanin. Since Teneurins engage in homophilic and heterophilic interactions that can mediate intercellular adhesion (Rubin et al., 2002; Boucard et al., 2014), they might facilitate the contact of platelets with tumor cells and promote tumor metastasis. The reported interaction of Teneurins with integrins seems consistent with such mechanism (Graner et al., 1998; Trzebiatowska et al., 2008), as specific integrin subtypes are expressed in tumor cells and on the platelet surface (Wright et al., 2011). A crossing point of Teneurins and integrins is supported by additional data. Hence, focal adhesion kinase (FAK)-dependent signaling was induced by Teneurin-4 in neuroblastoma cells, and phosphorylated FAK colocalized with Teneurin-4 at sites of neurite protrusion formation, together with the Rho GTPases cdc2 and Rac1 (Suzuki et al., 2014b). Integrin-mediated signaling commonly activates the FAK pathway, and inhibition of oncogenic FAK activity bears therapeutic importance (Kolev et al., 2017). Further, Rho GTPases are key regulators of cell migration (Sadok and Marshall, 2014) and have been implicated in several contexts as mediators of Teneurin functions (Nakaya et al., 2013; Glendining et al., 2017; Talamillo et al., 2017), as discussed above. In addition, the Teneurin-1 ICD was shown to bind to CAP/Ponsin (Nunes et al., 2005), a cytoskeleton adaptor molecule that interacts with FAK to regulate focal adhesion and cytoskeleton dynamics, thus impacting on cell adhesion and migration (Tomasovic et al., 2012). Finally, Teneurin-4 and Laminin, the common ligand for integrins, showed partly overlapping localization patterns in the developing avian gut (Kenzelmann-Broz et al., 2010), and in *C. elegans*, evidence suggested that laminin Epi-1 might act as a Ten-1 ligand (Trzebiatowska et al., 2008). Together, these findings suggest that Teneurins modulate and interact with components of integrin mediated signaling to modify crucial components required for cell migration and invasion. The localization of Teneurins at essential sites of cytoskeletal anchorage, focal adhesion, and attachment to the extracellular matrix, is consistent with this role. Teneurin-mediated adhesion is actively involved in cell signaling through well-characterized cancer-related pathways. Such signals might be initiated by context-dependent, hemophilic, or heterophilic intercellular contacts between Tenerins and/or integrins, or by Teneurin-mediated signaling derived from interaction with ECM components such as collagens.

The role of Teneurins as modulators of cytoskeleton dynamics might also be relevant to drug-resistance, where microtubules play an important part. As showed in *Drospohila*, Teneurin disruption lead to disorganized microtubule and α-spectrin-dependent cytoskeletal structures that impaired transsynaptic organization (Mosca et al., 2012), and in mouse hippocampal cells, TCAP-1 increased levels of tubulins alfa and beta at cellular protrusion sites (Chand et al., 2012). Further, the microtubule-actin cross-linking factor 1 (MACF1) protein was identified as a Teneurin-1-ICD binding protein (Schöler et al., 2015). Interestingly, interaction with MACF1 would strengthen the link of Teneurins with ERBB-mediated signaling, as in breast cancer cells, ß-heregulin could induce ERBB2-dependent protrusions that were enriched in microtubules (Zaoui et al., 2010). It could be shown that MACF1 acted as a downstream effector of ERBB2 signaling that mediated microtubule capture at the cell membrane. MACF1, which is highly expressed in the developing nervous system, is further required for WNT-signaling (Chen et al., 2006), whose involvement with Teneurins was discussed above. MACF1 expression was also prominent at advanced stages in brain tumors, including glioblastoma (Afghani et al., 2017). The authors showed that MACF1 knock-out could reduce proliferation and migration of glioblastoma cells, which was accompanied by a reduction in WNT signaling effectors. Further, downregulation of MACF1 increased sensitivity of glioblastoma cells to the DNA alkylating agent temozolomide. Since overexpression of the Teneurin-1-ICD increased resistance to this drug in glioblastoma (Talamillo et al., 2017), interaction of Teneurin-1 and MACF1 might contribute to this phenotype, possibly involving the stabilization of actin and microtubule cytoskeletal structures and WNT signaling activity. A role for Teneurins in drug resistance is also supported by the massive overexpression (> 200-fold) of Teneurin-2 in an ovarian cancer cell line resistant to vincristine, a microtubule-targeting vinca alkaloid (Buys et al., 2007). In addition to increases in transporter genes, Fibronectin-1 was also massively augmented, which lead the authors to propose a cell adhesion mediated drug resistant (CAM-DR) phenotype, in which anchorage to the ECM appears essential for cell survival in the presence of antineoplasic drugs. Interestingly, Lathrophilin-3 and several collagens were also upregulated, suggesting that Teneurins could interact in a common pathway with these adhesion molecules to mediate drug resistance related to adhesion. Consistent with this prediction, CAM-DR involves signaling through MEK/ERK and FAK pathways (Dickreuter and Cordes, 2017) and in breast cancer, upregulation of Teneurin-related Tenascin-C was implicated (Jansen et al., 2005). Also, the Ephrin receptor-A4 (EPHA4) was required for CAM-DR in multiple myeloma (Ding et al., 2017), and Teneurins are known regulators of Ephrin receptor expression in structures of the visual system (Young et al., 2013; Glendining et al., 2017). In this context, Teneurin-2 knock-out caused concomitant reductions in EPHB1 and CFOS, and the latter was also reduced upon Teneurin-3 knock-out (Merlin et al., 2013; Young et al., 2013). This suggests that expression of the oncogenic transcription factor c-Fos, which is a target of ERK-mediated phosphorylation, might be under transcriptional regulation of Teneurins. These findings again highlight the role of MEK-ERK signaling as a central component of Teneurin-mediated functions in tumors. The transcriptional regulation of Ephrin receptors, whose expression is frequently augmented in tumors, might provide a further mechanism of CAM-DR promotion through Teneurins.

In summary, altered expression of Teneurins has been demonstrated in numerous tumors and can be an early event in cell transformation. Recent data have provided new functional insights demonstrating that Teneurins respond-to and can orchestrate signaling pathways with known roles in carcinogenesis, invasion and drug resistance. Not surprisingly, Teneurin expression is associated to patient outcome and could bear prognostic implications. In future studies, distinct and domain-specific functions must be considered that involve Teneurin-mediated transcriptional regulation, extracellular signaling, and adhesion between cells and with components of the extracellular matrix. Consistent with this complex scenario, both up- and downregulation of Teneurins can be expected in tumors, and their dual tumor suppressive or oncogenic function might parallel that of other proteins implicated in cancer.

## Teneurins as Targets of Somatic Mutation

As evidenced in the above sections, tumor-related Teneurin alterations conform to mechanisms expected for typical cancer genes, which include changes in gene structure and expression levels suggestive of both oncogenic and tumor suppressive functions. If these predictions hold, it should be possible to identify additional mechanisms common to cancer genes, such as sequence changes through somatic mutation. The analysis of such findings presents some difficulties. First, Teneurins are encoded by very large genes. In probabilistic terms, this means that large DNA sequence stretches might be expected to show a proportionally high number of variants throughout the entire gene length, affecting different domains of the encoded protein. Second, functional studies to address the impact of every such change do not exist or are limited to different contexts, as will be discussed. Similarly, there is insufficient evidence to catalog single nucleotide variants as benign causes of genetic variation, or instead, as pathogenic somatic changes. In spite of these limitations, records of somatic alterations can be retrieved from large databases and subjected to a comparative analysis, as we expose below.

### The Impact of Single Nucleotide Variants

Considering the unknown functional impact of Teneurin SNVs in tumors, supportive evidence can be inferred from the analysis of variants in other pathogenic conditions. As mentioned, Teneurin genes are strongly conserved throughout species (Minet and Chiquet-Ehrismann, 2000; Tucker et al., 2012) and sequence variation might thus impact on protein function. Accordingly, a novel variant in *TENM3* was associated with developmental defects of the visual system leading to small eyes (microphtalmia) and impaired vision (Aldahmesh et al., 2012). The variant (p.T695Nfs^*^5) introduced a frameshift mutation resulting in a premature stop codon at the extracellular side of the Teneurin-3 transmembrane domain. It was shown to completely abolish *TENM3* gene expression, although the intracellular and transmembrane domains were correctly encoded. Since the variant was homozygously inherited in two affected siblings, it resulted in a null phenotype with complete absence of Teneurin-3. This phenotype is consistent with impairments in the visual system observed in Teneurin-3 knock-out mice (Leamey et al., 2007; Glendining et al., 2017). In terms of frequency, the mutation could not be found in healthy controls or in a database with variant coverage derived from >10.000 chromosomes, which supported it was an uncommon, pathogenic change. Some years later, 12 novel missense variants in *TENM4* were discovered in Spanish patients affected with essential tremor (Hor et al., 2015), a phenotype also observed in mice upon Teneurin-4 knock-out (Suzuki et al., 2012). Unlike microphtalmia, this condition was inherited in an autosomal dominant fashion, and eleven variants were predicted to be damaging by various *in silico* algorithms. Three variants were characterized further and showed a trend to altered Teneurin-4 protein clustering at cell membranes. Further, they increased the number of small-diameter neuronal axons and induced errors in branching and pathfinding in zebrafish, consistent with previous findings in Teneurin-4 knock-outs. The authors suggested a dominant-negative effect possibly related to an impaired ability of Teneurin-4 to engage in homophilic interactions, as two of the variants (p.T1367N, p.A1442T) altered essential NHL-repeat/β propeller motifs in the extracellular domain. In contrast, *TENM4* variants could not be associated with essential tremor in Canadian patients (Houle et al., 2017). In this cohort, missense variants were more frequent in cases (25%) than controls (14%), but could not be statistically associated with disease. One shortcoming was that variant segregation in cases and their relatives was not assessed. With few exceptions, missense changes were present in 1 or 2 individuals only, and nonsense variants were extremely rare with only one identified case. This suggests that deleterious loss of function changes are not well-tolerated in the germline and might be subjected to negative selection (Martincorena et al., 2017), while missense variants were more frequent but occurred at low allelic frequencies. The nonsense mutation generated a premature stop codon close to the Teneurin-4 C-terminus, which should preserve the largest portion of the protein intact. This bears similarity to a missense variant affecting a conserved residue within the globular C-terminal domain of murine Teneurin-4, which lead to embryonic failure due to severe gastrulation defects (Lossie et al., 2005). An additional missense variant within the Teneurin-3 C-terminal TCAP-domain was recently found to segregate with developmental dysplasia of the hip in a large pedigree with severe manifestations (Feldman et al., 2017). Interestingly, the cytogenetic localization of *TENM3* maps to a locus previously associated with this dysplastic condition, where at least one additional gene has been implicated (Zhao et al., 2017). These data suggest that, although a major part of the protein might not be affected, alterations at Teneurin C-terminal domains should not be dismissed in terms of potential phenotypic impact. Besides TCAPs, an apoptogenic C-terminal domain was recently described for Teneurin-2 (Ferralli et al., 2018). The physiologic stimuli triggering its release remain unknown, but the domain was proposed to model neural networks through selective induction of apoptosis at intersynaptic spaces. Finally, a rare missense variant in *TENM1* was associated with a familial case of congenital general anosmia (Alkelai et al., 2016). This variant (p.P1610L) had not been reported in the population and was categorized as damaging by eight predictive algorithms. According to current gene annotations, this change would localize amidst conserved tyrosine-aspartic acid (YD) repeat motifs. These repeats, best known from bacterial proteins, appear to be glycosylated and to mediate cellular aggregation (Feng et al., 2002; Rubin et al., 2002). Taken together, these reports demonstrate that SNVs in Teneurin genes can result in pathogenic outcomes, although the mechanisms involved have not been studied in depth. A potential functional redundancy between different Teneurins should also be considered, which might constrain the phenotypic severity derived from single pathogenic variants (Leamey et al., 2007; Trzebiatowska et al., 2008).

### Recurrent Somatic Changes in Lymphoma

The above examples of microphtalmia and essential tremor represent homozygous loss of function as opposed to heterozygous, dominant function gain, respectively. In cancer, such changes would match mechanisms of tumor suppressor loss and oncogene activation, triggered through acquisition of somatic mutations. Two studies addressing the mutational landscape in lymphomas have listed somatic variants in Teneurins. The first performed whole genome sequencing of diffuse large B-cell lymphoma, and could identify mutations in all Teneurin genes (Morin et al., 2013). Interestingly, *TENM2* was validated as frequently mutated in this cancer by analyzing data from additional cohorts. Further, *TENM2* mutations showed evidence for positive selection, which favored their driver function in tumorigenesis. Two of these mutations were nonsense and predicted the expression of a truncated protein or its degradation, in analogy to the variant observed in microphtalmia (Aldahmesh et al., 2012). However, it should be noted that most Teneurin variants in this report were accompanied by annotation errors and their precise location cannot be deduced. A second study performed whole-exome sequencing of primary lymphomas of the central nervous system (Vater et al., 2015). One missense mutation was found in *TENM3*, while *TENM4* was among the 4 most frequently mutated genes (missense mutations in 4 of 9 tumors). Three mutations mapped to the extracellular domain, of which two lied between YD repeats and one was predicted to affect an EGF-like motif. A fourth mutation affected a potential phosphorylation site within the ICD. These data suggest a potential functional impact for Teneurin mutations, which might contribute to tumorigenesis. The authors further noted that mutations in *TENM4* and *PIM1* were mutually exclusive. Interestingly, the oncogenic PIM1 serine-threonine kinase is essential for MYC-mediated tumorigenesis in triple-negative breast cancer cells, where knock-out of both genes was synthetic lethal (Horiuchi et al., 2016). Since Teneurin-1 did mediate MYC-RHOA-induced responses in glioblastoma (Talamillo et al., 2017), additional Teneurins might operate through a similar mechanism. Assuming that PIM1 and Teneurins both act through a MYC-dependent pathway, co-selection of mutations might not provide an additional advantage to tumor cells, explaining the lack of concurrent variants in these genes.

### The Mutation Profile of Teneurin Genes

**Figure 2:**
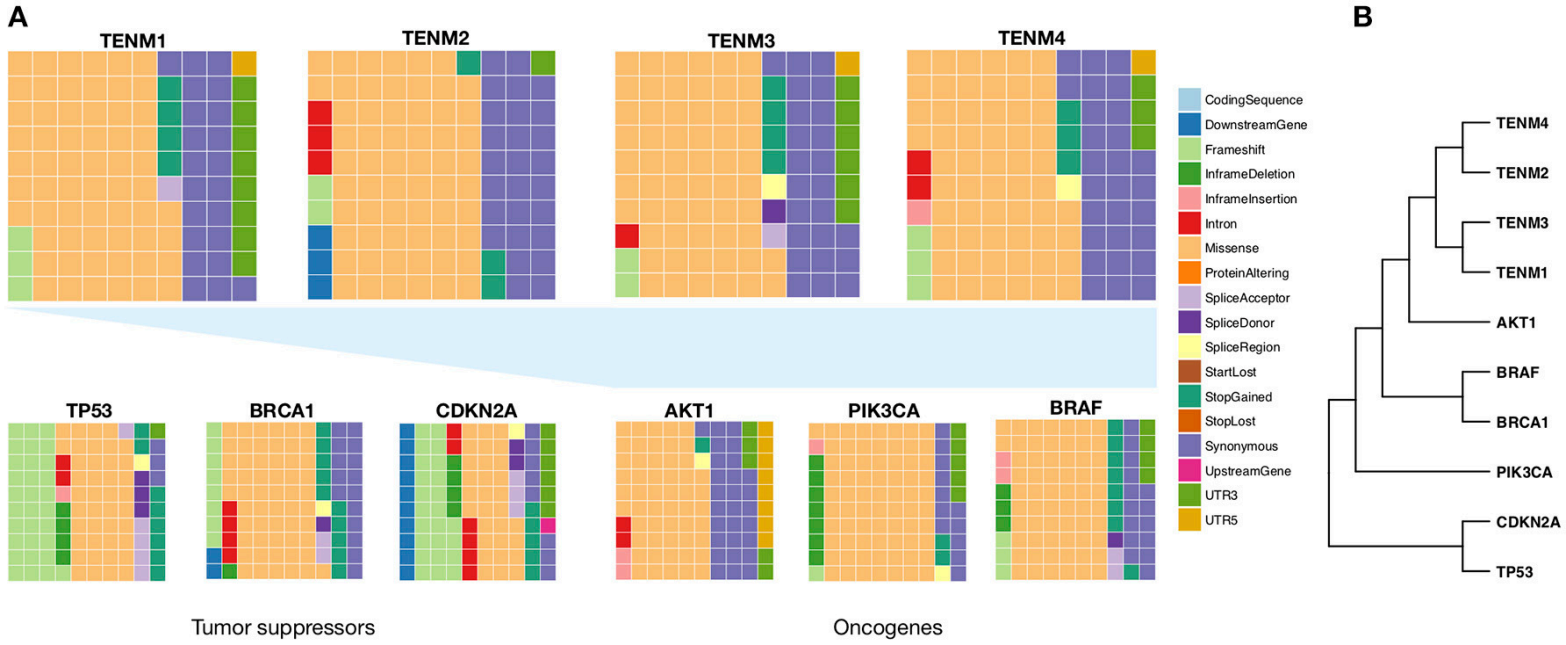
Frequency of mutation types targeting Teneurin genes. All somatic variants reported for Teneurin genes were retrieved from the Caner Genome Atlas, without distinction of tumor type. The number of variants was 1155 for *TENM1*, 777 for *TENM2*, 938 for *TENM3*, and 772 for *TENM4*. For the remaining genes, variant numbers were 97 for *AKT1*, 212 for *BRAF*, 385 for *BRCA1*, 346 for *CDKN2A*, 404 for *PIK3CA*, and 1000 for *TP53*. **(A)** Waffle charts describing the proportion of each mutation category. Each square represents a frequency of 1%. Waffle charts at the bottom were calculated from TCGA through the same procedure and are included for comparison. **(B)** Euclidean-distance cladogram grouping genes based on their mutation type frequency distribution was constructed using the R package *hclust*.

Based on the above data, Teneurins appear to be a frequent target for somatic mutation in lymphomas, and the phenotypic consequence of singe nucleotide changes is evidenced from genetic conditions associated with germline changes. To assess if similar findings apply to other tumors, we performed gene-based searches for all Teneurins in The Cancer Genome Atlas (TCGA, https://cancergenome.nih.gov/). Teneurin mutations recorded for the entire TCGA cohort are represented in Figure 2A, sorted according to their subtype. The mutation spectrum of known oncogenes (*AKT1, PIK3CA, BRAF*) and tumor suppressor genes (*TP53, BRCA1, CDKN2A*) is included for comparison. Over 750 variants could be retrieved for each Teneurin gene. As evidenced by the graphs, Teneurin missense changes clearly predominate and their mutation spectra do not resemble those of TP53 or CDKN2A. Nonsense variants were infrequent, in analogy to germline findings in essential tremor patients (Houle et al., 2017). When the mutation subtype frequency was represented in a Euclidean-distance cladogram (Figure 2B), Teneurin genes clustered together and their mutation profiles were closer to that of AKT1. However, the next association level was with BRAF and BRCA1, an oncogene and tumor suppressor gene, respectively. According to this distribution, the mutation spectrum of Teneurins places them closer to oncogenes, but a less frequent tumor suppressive pattern cannot be excluded. Functional assessment will be required to assign a phenotypic impact to each variant, in addition to predictive algorithms of pathogenic potential as those applied for heritable conditions. An open question concerns the presence of frequent synonymous variants in the germline (Houle et al., 2017) and in tumors, assumed to represent genetic polymorphisms with restrained phenotypic effects. However, a recent bioinformatics analysis suggested that oncogenes, but not tumor suppressors, are affected by an excess of synonymous somatic mutations in tumors (Supek et al., 2014). Further, selection of synonymous mutations was gene and tumor-specific and targeted evolutionary conserved residues. The data suggested that synonymous variants could alter exonic splicing elements, leading to differential exon usage. As reported recurrently, Teneurins are subjected to diverse alternative splicing patterns throughout species (Tucker et al., 2001, 2012; Lossie et al., 2005; Silva et al., 2011; Graumann et al., 2017; Berns et al., 2018; Jackson et al., 2018; Li et al., 2018a), and the sequences that define the splicing system might be altered by missense but also synonymous changes. If applied to Teneurins, the reported results appear to favor an oncogenic role driven by missense and perhaps synonymous single nucleotide mutations.

## Concluding Remarks

In recent investigations, exciting discoveries have revealed the amazing level of complexity that surrounds the function of Teneurins. These large proteins present different domains enabling a range of biological functions from adhesion to cell signaling. As could be predicted form their evolutionary conservation, genetic changes are demonstrating phenotypic consequences that underlie different heritable conditions. Reviewing the available literature and retrieving information from cancer databases, we could now gather evidence that strongly supports a role of Teneurins in tumorigenesis. Clearly, these genes are affected by tumor-specific changes through mechanisms expected for validated cancer genes, including chromosomal alterations, somatic mutations, and aberrant expression patterns. The inherent function of Teneurins as signaling molecules consistently involves well-established cancer pathways, such as NOTCH, WNT, and the NRG/ERBB axis. Further, their essential function as adherence molecules can alter processes related to cell adhesion, migration and invasion, and impact on cell plasticity by modulating cell differentiation and transition between epithelial and mesenchymal phenotypes. Accordingly, a prognostic association of Teneurin expression with patient survival has been demonstrated for various cancer types. Based on current molecular evidence, it seems highly probable that Teneurins might exhibit an oncogenic contribution to tumor initiation, growth and progression, although a dual function including tumor suppressive roles cannot be excluded at present. A major issue will be the validation of the pathogenic impact of Teneurin somatic changes, which will be challenging considering Teneurin gene lengths, their complex pattern of alternatively spliced species, and their tissue-specific relevance. The number of somatic variants in tumors is large and most seem to occur at low allele frequencies. The rising volume of omics data available through patient-based repositories will indubitably provide an important means to address some of these challenges, and aid to the validation of Teneurins as representative cancer genes.

## Author Contributions

AZ performed literature and data searches, retrieved data from repositories, created tables, and wrote the manuscript. BR-J retrieved and analyzed data from repositories, created figures, and critically reviewed the manuscript.

### Conflict of Interest Statement

The authors declare that the research was conducted in the absence of any commercial or financial relationships that could be construed as a potential conflict of interest.
